# Cardiovascular and lifestyle risk factors of mild cognitive impairment in UK veterans and non-veterans

**DOI:** 10.1093/occmed/kqae027

**Published:** 2024-06-11

**Authors:** R Akhanemhe, S A M Stevelink, A Corbett, C Ballard, H Brooker, B Creese, Dag Aarsland, Adam Hampshire, Neil Greenberg

**Affiliations:** King’s Centre for Military Health Research, Department of Psychological Medicine, Institute for Psychiatry, Psychology & Neuroscience, King’s College London, London, UK; King’s Centre for Military Health Research, Department of Psychological Medicine, Institute for Psychiatry, Psychology & Neuroscience, King’s College London, London, UK; Department of Psychological Medicine, Institute for Psychiatry, Psychology & Neuroscience, King’s College London, London, UK; Exeter University Medical School, University of Exeter, Exeter, UK; Exeter University Medical School, University of Exeter, Exeter, UK; Exeter University Medical School, University of Exeter, Exeter, UK; Division of Psychology, Department of Life Sciences, Brunel University London, London, UK; Department of Old Age Psychiatry, Institute of Psychiatry, Psychology, and Neuroscience, King’s College London, London, UK; Department of Medicine, Imperial College London, London, UK; King’s Centre for Military Health Research, Department of Psychological Medicine, Institute for Psychiatry, Psychology & Neuroscience, King’s College London, London, UK

## Abstract

**Background:**

The link between poor cardiovascular health (CVH), lifestyle and mild cognitive impairment (MCI) has been well established in the general population. However, there is limited research exploring these associations in ageing UK veterans.

**Aims:**

This study explored the risk of MCI and its association with nine CVH and lifestyle risk factors (including diabetes, heart disease, high cholesterol, high blood pressure, obesity, stroke, physical inactivity, the frequency of alcohol consumption and smoking) in UK veterans and non-veterans.

**Methods:**

This prospective cohort study comprised data from the PROTECT study between 2014 and 2022. Participants comprised of UK military veterans and non-veterans aged ≥50 years at baseline. Veteran status was defined using the Military Service History Questionnaire. CVH and lifestyle risk factors were defined using a combination of self-report measures, medication history or physical measurements. MCI was defined as the presence of subjective and objective cognitive impairment.

**Results:**

Based on a sample of 9378 veterans (*n* = 488) and non-veterans (*n* = 8890), the findings showed the risk of MCI significantly reduced in veterans with obesity, those who frequently consumed alcohol and were physically inactive compared to non-veterans. The risk of MCI significantly increased in veterans with diabetes (hazards ratio [HR] = 2.22, 95% confidence interval [CI] 1.04–4.75, *P* ≤ 0.05) or high cholesterol (HR = 3.11, 95% CI 1.64–5.87, *P* ≤ 0.05) compared to veterans without.

**Conclusions:**

This study identified CVH and lifestyle factors of MCI in UK veterans and non-veterans. Further work is needed to understand these associations and the underpinning mechanisms which could determine intervention strategies to reduce the risk of MCI.

## Teaser

Research has shown cardiovascular health (CVH) and lifestyle risk factors increase the risk of mild cognitive impairment (MCI) in the ageing general population. However, this information is limited in the ageing veteran population. As no studies have compared the risk of MCI between veterans and non-veterans in the UK, this presents an opportunity to conduct the first comparative study using a UK cohort of 9378 individuals aged 50 years and over.

**Key learning points**

**What is already known about this subject:**

- Previous evidence in the general population has shown exposure to cardiovascular health, or lifestyle choices, are modifiable risk factors for mild cognitive impairment.
- This is concerning as veterans are at an increased risk of cardiovascular health or lifestyle factors. This could potentially have a negative consequence on their cognitive function with age.

**What this study adds:**

- This is the first study to quantify the risk of mild cognitive impairment in UK veterans exposed to nine key cardiovascular health and lifestyle risk factors compared to the non-veteran population using longitudinal data.
- Obesity, alcohol consumption and physical inactivity reduced the risk of mild cognitive impairment in veterans compared to non-veterans.
- Within the veteran sample, diabetes and high cholesterol increased the risk of mild cognitive impairment.

**What impact this may have on practice or policy:**

- Early diagnosis and intervention of modifiable risk factors may reduce the risk of mild cognitive impairment and subsequently dementia in both veterans and non-veterans.

## INTRODUCTION

The number of individuals living with mild cognitive impairment (MCI) is due to increase because of an increasing ageing population; this presents a potentially very large public health challenge [1]. It has been proposed that a constellation of modifiable CVH and lifestyle risk factors (including high blood pressure, high cholesterol, heart disease, type 2 diabetes, stroke, obesity, cigarette smoking, alcohol consumption and physical inactivity) could potentially prevent and manage MCI and dementia [2] as poor CVH and lifestyle factors are highly attributable to age-related cognitive decline and dementia [3].

As there are limited treatments available for MCI [1,2], prevention through managing modifiable CVH and lifestyle risk factors could play an integral role in (1) reducing the prevalence of MCI and dementia [3], (2) providing an alternative approach to slowing down cognitive ageing and promote successful ageing [4], and (3) acting as a major preventative measure and therapeutic target against cognitive decline and dementia [3–5].

Previous research has investigated the association between this constellation of risk factors and MCI, or cognitive deficits based on the general population [6]. However, little is known about this association in individuals with a history of serving in the Armed Forces. Generally, recruitment into the Armed Forces requires a pre-service health assessment to identify one has good physical health (including cardiovascular) and personnel are expected to adopt and maintain a healthy lifestyle during their service [7]. However, during transition back into civilian life post-service, evidence suggests there is a significant decrease in the maintenance of a healthy lifestyle amongst veterans [8]. This post-service lifestyle may increase the risk that veterans will develop a CVH condition. For instance, one study found the rate of obesity was higher in veterans compared to the general population which was instigated by key factors including mental ill health [9].

Prior research has found that, compared to those who had never been in the military, US veterans had higher rates of cardiovascular disease [10]. Data from the UK have also shown that veterans are more likely to have smoked cigarettes compared to the general population (55% versus 44%) [11]. Unsurprisingly, smoking has been shown to increase the risk of MCI which has been linked to neurobiological changes including a reduced entorhinal cortex [12]. Moreover, veterans were less likely to follow the recommended amount of exercise post-service which could increase the risk of cognitive decline and dementia [13,14].

Research has independently investigated the relationship between CVH or lifestyle risk factors, including alcohol consumption, high blood pressure, high cholesterol, obesity, smoking, physical inactivity, stroke, heart disease or diabetes, and MCI or dementia in the general population [2]. However, little is known about the relationship between this constellation of risk factors and MCI in military veterans. Therefore, this study aimed to (1) investigate if the risk of MCI differed between veterans and non-veterans living with a CVH or lifestyle risk factor and (2) investigate if the risk of MCI differed between veterans living with or without a CVH or lifestyle risk factor.

## METHODS

We used data from the Platform for Research Online to investigate Genetics and Cognition in Ageing (PROTECT) study, a prospective cohort study that aims to understand risk factors associated with cognitive decline and MCI in the ageing population in the UK. Full details of the PROTECT study are published elsewhere [15]. Approximately, 30 760 participants took part in the PROTECT study at baseline between 2014 and 2022. The eligibility criteria for this study were as follows: aged 50 years and over at baseline, do not fit the criteria for MCI during the baseline period, no diagnosis of dementia or any neurodegenerative disorder, and completed the main PROTECT study and the nested cross-sectional military study.

Participants were recruited into the PROTECT study between 2014 and 2022 through charities, social media and the NHS, and were asked to complete assessments annually using an online platform. For the nested military study, veterans were recruited through military charities in 2019 and were asked to complete assessments once. As part of the data cleaning process, data from the main PROTECT study and the nested military study were matched based on subject ID. For this study, participant data were excluded if they did not complete both the main PROTECT study and the nested military study and had MCI at baseline.

Veteran status was the primary independent variable which was defined using the Military Service History Questionnaire (Supplementary A, available as Supplementary data at *Occupational Medicine* Online) which was structured similarly to questionnaires used in previous research [16]. Participants were stratified as veterans [17] and non-veterans. The second independent variable comprised nine individual CVH and lifestyle risk factors. CVH risk factors included (1) hypertension (self-reported endorsed as yes or taking anti-hypertensives), (2) obesity was calculated based on self-reported weight (kilograms) and height (metre; body mass index [BMI] ≥30 kg/m^2^) [18], (3) high cholesterol (self-reported endorsed as yes or taking anti-cholesterol medication), (4) diabetes (self-reported endorsed as yes), (5) stroke (self-reported endorsed as yes) and (6) heart disease (self-reported endorsed as yes). Lifestyle risk factors included (1) physical activity (self-reported endorsed as inactive), (2) current cigarette smoker (self-reported endorsed as yes) and (3) frequent alcohol consumption (self-reported endorsed as frequent consumption).

MCI was the outcome of this study. Participants were asked to complete MCI assessments at each time point. MCI was defined similarly to the International Working Group [19]. This was defined as (1) subjective cognitive decline with an average score ≥3.01 on the Informant Questionnaire on Cognitive Decline in the. Elderly (IQCODE) [20] and (2) objective cognitive decline with a performance ≥1 standard deviation below the mean in either the digit span, self-ordered search, verbal reasoning, the paired associate learning test, the switching Stroop test part A or the trails making test part B [21]. If participants fit the criteria, they were classified as MCI, and those who did not fit the criteria were classified as cognitively normal.

We obtained additional data to be used as covariates. (1) Socio-demographic: gender (male, female), education level (secondary, post-secondary, vocational, university), ethnicity (White, ethnic minorities), marital status (living in a relationship [married, civil partnership, co-habiting], was previously in a relationship [divorced, widowed, separated], single), and employment status (employed, retired, unemployed). (2) Any mental disorder where participants classed as AMD if they had caseness for probable PTSD (6-item PTSD checklist [22] with a score ≥13 for caseness), probable anxiety disorder (the 7-item Generalized Anxiety Disorders [23] Questionnaire with a score ≥7 for caseness), or probable depression (9-item Patient Health Questionnaire [24] with a cut-off score ≥7). (3) Family history of dementia (FHD) was self-reported. If participants responded yes to having a first-degree relative with any of the subtypes of dementia, they were classed as family history present, and if they responded no, they were classed as family history absent. (4) Military service history variables were derived from the MHSQ which included: duration of service (<4 years, ≥4 years), deployment history (yes or no), type of service (Regular, Reservist, both, other) and last rank held. Last rank was divided into Commissioned officer, Private or Non-Commissioned officer, and other.

For the statistical analyses, baseline socio-demographic, clinical and military characteristics were summarized using proportions for the overall sample and then stratified by veteran status (veterans versus non-veterans). The relationship between each characteristic variable and veteran status was assessed using Chi-square or Fisher’s exact test. For all the longitudinal analyses, the follow-up time was calculated from the baseline date until the date MCI occurred. The time scale was defined by age (in years). Cox proportional hazard regression (hereby referred to as cox regression) was used to compare the risk of MCI in two separate models. (A) This first model compared the risk of MCI in non-veterans (reference category) and veterans with each CVH and lifestyle risk factor at baseline. (B) The second model only focused on the veteran sample to compare the risk of MCI between veterans without each CVH or lifestyle risk factor at baseline (reference category) to veterans with each CVH or lifestyle risk factor at baseline.

Participants were censored if they did not fit the criteria for MCI by the end of the study or were lost to follow-up. This study included panel data, so cox regression was clustered by subject ID to account for multiple records using robust standard errors. The survival rates were graphically displayed using survival probability plots. A series of independent adjusted cox regression analyses were conducted adjusting for covariates. This included socio-demographics, mental health factors, FHD and military service history (in model B only). These were priori-selected covariates. The hazard ratio (HR) was presented for the unadjusted models, the adjusted hazard ratio (aHR) for the adjusted models and 95% confidence intervals (CIs) were reported. To ensure the models met the assumptions for cox regression, proportionality was verified by Schoenfeld residuals (*P* > 0.05).

All statistical analyses were conducted using STATA, version 17.0, and a significant threshold of *p* ≤ 0.05 was used in all of the analyses.

## RESULTS

The total sample size at baseline was 9378 comprising 488 (5.2%) veterans and 8890 (94.8%) non-veterans. Table 1 summarizes the baseline characteristics of the study population. A greater proportion of the veteran sample were in the 50–64 years age group (48%), were males (61%), retired (61%) and educated to a university level (45%). Under one-quarter of the overall sample had high blood pressure (23%). The prevalence of obesity in the overall sample was 15%. Under one-quarter of the overall sample were physically inactive (23%). Over two-thirds of the sample frequently consumed alcohol (67%).

**Table 1.** Baseline characteristics in the overall sample and by veteran status

	Overall sample (*n* = 9378), *n* (%)	Veteran (*n* = 488), *n* (%)	Non-veteran (*n* = 8890), *n* (%)	*P*-value
Age group				<0.05^*^
50–64 years	6117 (65)	235 (48)	5882 (66)	
65–79 years	2902 (31)	214 (44)	2688 (30)	
**≥**80 years	359 (4)	39 (8)	320 (4)	
Gender, males	2188 (24)	292 (61)	1896 (22)	<0.05^*^
Education level				<0.05^*^
Secondary	1052 (11)	84 (18)	968 (11)	
Post-secondary	1016 (11)	58 (12)	958 (11)	
Vocational	1791 (20)	118 (25)	1673 (19)	
University	5308 (58)	216 (45)	5092 (59)	
Employment				<0.05^*^
Employed	4246 (46)	176 (37)	4070 (47)	
Retired	4642 (51)	292 (61)	4350 (50)	
Unemployed	275 (3)	8 (2)	267 (3)	
Marital status, Single	597 (7)	24 (5)	573 (7)	NS
Ethnicity, White	9011 (98)	469 (98)	8542 (98)	NS
Cigarette smoker, positive	233 (3)	12 (3)	221 (3)	NS
Physically inactive, positive	2104 (23)	93 (19)	2011 (23)	NS
Alcohol frequency				<0.05^*^
Never	602 (6)	38 (8)	564 (6)	
Less than once a week or month	2512 (27)	107 (22)	2405 (27)	
Weekly/frequently	6252 (67)	342 (70)	5910 (67)	
Diabetes, positive	280 (3)	22 (5)	258 (3)	<0.05^*^
Stroke, positive	103 (1)	5 (1)	98 (1)	NS^a^
High blood pressure, positive	2132 (23)	147 (30)	1985 (22)	<0.05^*^
High cholesterol, positive	637 (7)	40 (8)	597 (7)	NS
Obesity, positive	1430 (15)	74 (15)	1356 (15)	NS
Heart disease, positive	370 (4)	44 (9)	326 (4)	<0.05^*^
AMD caseness	1041 (11)	50 (10)	991 (11)	NS
FHD, present	3220 (43)	136 (36)	3084 (43)	<0.05^*^
Duration of service, >4 years		288 (59)		NC
Branch				NC
Naval services		128 (26)		
British Army		202 (41)		
Royal Air Force		158 (33)		
Type of engagement				NC
Regular		288 (64)		
Reservist		105 (23)		
Both		56 (13)		
Deployment history (yes)		170 (35)		
Lank rank				NC
Private or Non-Commissioned Officer		275 (57)		
Officer		186 (38)		
Other		26 (5)		

AMD, any mental disorder; FHD, family history of dementia; NC, not calculated, NS, non-significant.

This table presents the column percentages of each characteristic in the overall sample and then stratified by veteran status (veteran versus non-veteran). In the stratified data (veteran versus non-veteran), the portions presented were within each group.

Numbers may not add up due to missing data.

^a^Calculated using Fisher test; Percentages rounded up to the nearest whole numbers.

^*^Significant at *P* ≤ 0.05.

**Figure 1.**
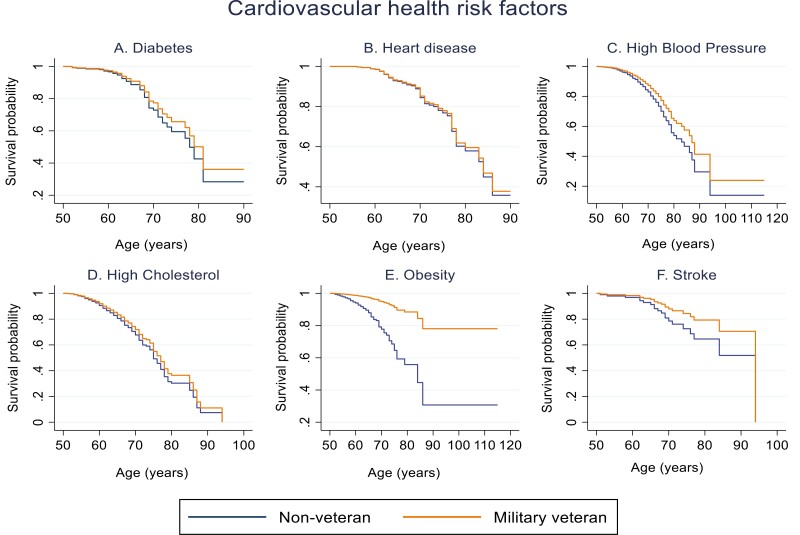
Survival probability graphs of MCI risk (unadjusted models) between non-veterans (reference category) and military veterans with each CVH risk factor: (A) diabetes, (B) heart disease, (C) high blood pressure, (D) high cholesterol, (E) obesity, (F) stroke.

**Figure 2.**
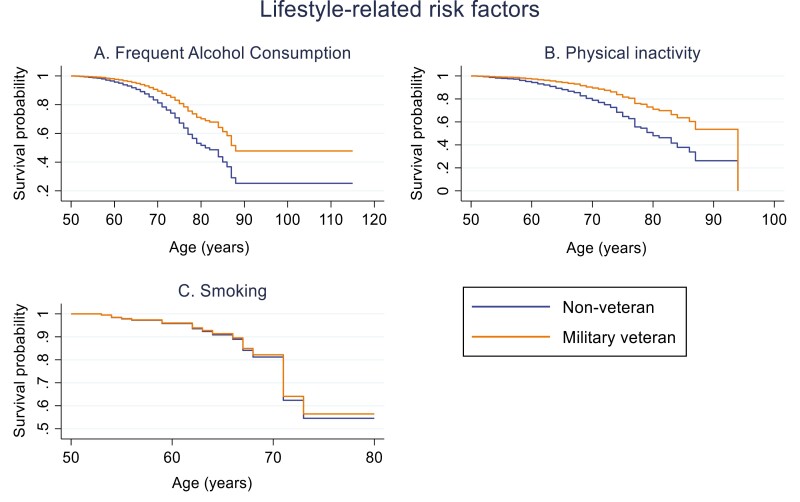
Survival probability graphs of MCI risk (unadjusted models) between non-veterans (reference category) and military veterans with each lifestyle-related risk factor: (A) frequent alcohol consumption, (B) physical inactivity, (C) smoking.

Model A compared the risk of MCI between veterans and non-veterans. The risk of MCI was significantly reduced in veterans with obesity (HR = 0.21, 95% CI 0.07–0.65, *P* ≤ 0.05), who were frequent alcohol consumers (HR = 0.53, 95% CI 0.38–0.74, *P* ≤ 0.05) and were physically inactive (HR = 0.46, 95% CI 0.25–0.85, *P* ≤ 0.05) compared to non-veterans. There was no significant difference in the risk of MCI between veterans and non-veterans with diabetes, heart disease, high blood pressure, high cholesterol, stroke or were smokers (see Table 2). A recurring trend was observed in the survival probability graphs for CVH risk factors (see Figure 1) and lifestyle risk factors (see Figure 2). The probability of survival decreased as age increased and declined faster in non-veterans but the difference to veterans was minimal.

**Table 2.** Unadjusted and adjusted risk of MCI in veterans and non-veterans with CVH and lifestyle risk factors

	Unadjusted model	Adjusted models		
		Model 1	Model 2	Model 3
	HR (95% CI)	aHR (95% CI)	aHR (95% CI)	aHR (95% CI)
Diabetes (*n* = 280)				
Non-veterans	1 (Reference)	1 (Reference)	1 (Reference)	1 (Reference)
Veterans	0.81 (0.37-1.76)	0.61 (0.23-1.61)	0.88 (0.38-2.02)	0.80 (0.38-1.68)
Heart disease (*n* = 370)				
Non-veterans	1 (Reference)	1 (Reference)	1 (Reference)	1 (Reference)
Veterans	0.95 (0.49-1.81)	0.82 (0.41-1.65)	0.64 (0.29-1.39)	0.92 (0.48-1.77)
High blood pressure (*n* = 2132)				
Non-veterans	1 (Reference)	1 (Reference)	1 (Reference)	1 (Reference)
Veterans	0.72 (0.48-1.08)	0.76 (0.49-1.17)	0.73 (0.48-1.10)	0.72 (0.48-1.08)
High cholesterol (*n* = 637)				
Non-veterans	1 (Reference)	1 (Reference)	1 (Reference)	1 (Reference)
Veterans	0.84 (0.48-1.47)	0.78 (0.44-1.39)	0.82 (0.46-1.44)	0.82 (0.47-1.43)
Obesity (*n* = 1430)				
Non-veterans	1 (Reference)	1 (Reference)	1 (Reference)	1 (Reference)
Veterans	0.21 (0.07-0.65)^*^	0.23 (0.07-0.74)^*^	0.20 (0.06-0.66)^*^	0.19 (0.06-0.62)^*^
Stroke (*n* = 103)				
Non-veterans	1 (Reference)	1 (Reference)	1 (Reference)	1 (Reference)
Veterans	0.53 (0.08-3.33)	0.31 (0.09-1.04)	0.51 (0.05-4.89)	0.52 (0.11-2.58)
Alcohol consumption (*n* = 2512)				
Non-veterans	1 (Reference)	1 (Reference)	1 (Reference)	1 (Reference)
Veterans	0.53 (0.38-0.74)^*^	0.63 (0.44-0.90)^*^	0.54 (0.38-0.76)^*^	0.53 (0.37-0.74)^*^
Physical inactivity (*n* = 2104)				
Non-veterans	1 (Reference)	1 (Reference)	1 (Reference)	1 (Reference)
Veterans	0.46 (0.25-0.85)^*^	0.48 (0.25-0.90)^*^	0.47 (0.25-0.87)^*^	0.44 (0.24-0.82)^*^
Smoking (*n* = 233)				
Non-veterans	1 (Reference)	1 (Reference)	1 (Reference)	1 (Reference)
Veterans	0.94 (0.09-9.35)	2.41 (0.34-16.72)	1.02 (0.10-9.89)	0.95 (0.09-9.98)

The *N* values presented for each risk factor is the total sample size at baseline used in each Cox model.

Model 1: Adjusted for demographics (gender, education, marital status, ethnicity, employment status).

Model 2: Adjusted for comorbid mental health (AMD: depression, PTSD or anxiety disorder).

Model 3: Adjusted for family history of dementia.

^*^Significant findings where *P* ≤ 0.05.

Model B explored the risk of MCI in the veteran sample. The risk of MCI significantly increased in veterans with diabetes (HR = 2.22, 95% CI 1.04–4.75, *P* ≤ 0.05) or high cholesterol (HR = 3.11, 95% CI 1.64–5.87, *P* ≤ 0.05) compared to veterans without. There was no significant difference in the risk of MCI between veterans with or without the following risk factors: heart disease, high blood pressure, obesity, stroke, frequent alcohol consumption, physical inactivity and smoking (see Table 3).

**Table 3.** Unadjusted and adjusted risk of MCI in veterans with and without CVH and lifestyle risk factors

	Unadjusted model	Adjusted models			
		Model 1	Model 2	Model 3	Model 4
	HR (95% CI)	aHR (95% CI)	aHR (95% CI)	aHR (95% CI)	aHR (95% CI)
Diabetes (*n* = 488)					
Veterans (−)	1 (Reference)	1 (Reference)	1 (Reference)	1 (Reference)	1 (Reference)
Veterans (+)	2.22 (1.04–4.75)^*^	2.71 (1.24–5.93)^*^	1.94 (0.81–4.63)	2.22 (1.03–4.77)^*^	1.83 (0.75–4.49)
Heart disease (*n* = 488)					
Veterans (−)	1 (Reference)	1 (Reference)	1 (Reference)	1 (Reference)	1 (Reference)
Veterans (+)	1.37 (0.72–2.59)	1.53 (0.76–3.06)	1.11 (0.55–2.24)	1.36 (0.71–2.60)	1.35 (0.67–2.74)
High Blood Pressure (*n* = 488)					
Veterans (−)	1 (Reference)	1 (Reference)	1 (Reference)	1 (Reference)	1 (Reference)
Veterans (+)	1.19 (0.70–2.02)	1.44 (0.84–2.49)	1.12 (0.63–1.96)	1.18 (0.70–2.01)	1.18 (0.64–2.18)
High Cholesterol (*n* = 488)					
Veterans (−)	1 (Reference)	1 (Reference)	1 (Reference)	1 (Reference)	1 (Reference)
Veterans (+)	3.11 (1.64–5.87)^*^	3.02 (1.53–5.96)^*^	2.79 (1.41–5.52)^*^	3.10 (1.64–5.86)^*^	2.06 (0.92–4.55)
Obesity (*n* = 488)					
Veterans (−)	1 (Reference)	1 (Reference)	1 (Reference)	1 (Reference)	1 (Reference)
Veterans (+)	0.38 (0.11–1.24)	0.27 (0.07–1.01)	0.29 (0.08–1.05)	0.38 (0.12–1.25)	0.41 (0.12–1.33)
Stroke (*n* = 488)					
Veterans (−)	1 (Reference)	1 (Reference)	1 (Reference)	1 (Reference)	1 (Reference)
Veterans (+)	0.35 (0.04–3.58)	0.23 (0.02–3.47)	0.26 (0.02–3.48)	0.35 (0.03–3.68)	1.07 (0.25–4.58)
Alcohol consumption (*n* = 487)					
Veterans (−)	1 (Reference)	1 (Reference)	1 (Reference)	1 (Reference)	1 (Reference)
Veterans (+)	1.23 (0.42–3.63)	1.95 (0.47–8.03)	0.98 (0.34–2.78)	1.28 (0.42–3.86)	1.01 (0.36–2.78)
Physical inactivity (*n* = 487)					
Veterans (−)	1 (Reference)	1 (Reference)	1 (Reference)	1 (Reference)	1 (Reference)
Veterans (+)	0.73 (0.35–1.51)	0.61 (0.27–1.36)	0.62 (0.27–1.40)	0.73 (0.35–1.54)	0.65 (0.30–1.41)
Smoking (*n* = 487)					
Veterans (−)	1 (Reference)	1 (Reference)	1 (Reference)	1 (Reference)	1 (Reference)
Veterans (+)	1.89 (0.19–18.32)	1.57 (0.19–13.01)	2.07 (0.22–19.22)	1.89 (0.19–18.48)	1.89 (0.18–19.88)

(−) Veterans without risk factor, (+) veterans with risk factor.

The *N* values presented for each risk factor is the total sample size at baseline used in each Cox model out of the total data available in the military veteran sample (*n* = 488).

Model 1: Adjusted for demographics (gender, education, marital status, ethnicity, employment status).

Model 2: Adjusted for comorbid mental health (AMD: depression, PTSD or anxiety disorder).

Model 3: Adjusted for family history of dementia.

Model 4: Adjusted for military service history (duration of service, branch of service, type of service, deployment history, last rank).

^*^Significant findings where *P* ≤ 0.05.

Post-estimation showed the risk of MCI was proportional (*P* > 0.05) in all unadjusted and adjusted models. Therefore, the assumptions were met in all the models.

## DISCUSSION

This prospective cohort study investigated the association between a constellation of CVH and lifestyle risk factors and MCI in UK veterans and non-veterans. From this, there were two main findings: (1) The risk of MCI was significantly reduced in veterans with obesity, who were frequent alcohol consumers and physically inactive compared to non-veterans. (2) Within the veteran sample, diabetes and high cholesterol significantly increased the risk of MCI.

The Healthy Worker Effect (HWE) proposed individuals in a specific type of employment are expected to exhibit lower levels of mortality or morbidity as those who are unwell are likely to enter this type of employment [25]. This applies to the military population as they were recruited into the Armed Forces based on good physical health, and any illnesses are likely to be detected earlier by the UK Defence Medical Services. Therefore, due to these health-protective measures, the risk of MCI is expected to be lower in the military population compared to the general population. This was reflected in some of the findings in this study as the risk of MCI was reduced in veterans who frequently consumed alcohol, were obese or physically inactive compared to non-veterans even though the proportions of these factors between veterans and non-veterans were comparable. For alcohol consumption, there is an unexplained variable that increased the resilience against MCI in veterans which requires further investigation. For physical inactivity there have been studies that found exercise at young and middle adulthood has a positive effect on cognition and neurobiological mechanisms [26,27]. Individuals can join the Armed Forces from the age of 16 years in the UK [28] and as frequent exercise and intensive training are a requirement of the Armed Forces, it is plausible engaging in these activities at a young age could have a protective effect against MCI in late life, but this also requires further investigation. Obesity in this study was defined using BMI which has its limitations. BMI does not distinguish between fat, muscle and bone density [29] so it is possible veterans could have fit the criteria of obesity, but not due to high body fat but other biological factors.

The relationship between diabetes, high cholesterol and MCI identified within the veteran sample aligns with previous research that explored this association in the general population [30,31]. Diabetes is associated with glucose-mediated toxicity and both diabetes and high cholesterol result in cerebrovascular abnormalities which could result in neurodegeneration and cognitive deficits [2,30,31]. Although there are no studies that have explored the HWE in veterans with diabetes or high cholesterol, these findings are supported by a US study that found the HWE wanes in veterans with cardiovascular conditions that are connected to diabetes and high cholesterol which could explain the increased MCI risk [10,30–34].

Caution is required when applying the HWE to the UK veteran population. Our findings showed the HWE wanes with high cholesterol or diabetes but remains stable with frequent alcohol consumption, obesity, and physical inactivity. This suggests the HWE against MCI is not specific to all CVH or lifestyle risk factors.

There are several benefits to the online data collection method used in the PROTECT study. Conducting online research increased accessibility irrespective of the geographical location, there was no need to travel to the research site hence significantly reducing the funds and time spent on travelling, especially for participants with mobility challenges. This meant there were increased opportunities amongst the ageing community to participate in this study.

This study also had notable limitations. First, with many risk factors, this study did not use a cardiovascular risk score such as the Cardiovascular Risk Factors, Aging and Dementia [28]. These risk indices are beneficial in clustering the level of the effect (as low, moderate or high) of CVH and lifestyle risk factors so there is a clear understanding of its relationship with MCI and dementia. However, using a risk score at the current stage of this project would limit the understanding of each individual risk factor. Future research should consider statistical tests such as factor or latent class analysis to identify classes for comorbidities. Second, self-reported or medication history was used to identify certain risk factors which was potentially unreliable opposed to using traditional clinical measures such as glucose, or sphygmomanometer readings which were unavailable. Third, there was bias in the sampling and recruitment method used in the PROTECT study as participants were asked to complete the study online. The PROTECT study may have recruited individuals who were highly familiar and confident with technology. This likely impacted the external validity as the whole ageing population were not represented by not capturing older adults who struggle with technology.

As the relationship between CVH, lifestyle risk factors and MCI is biological in nature, it is vital to explore the biological mechanisms. Future research should explore the neural correlates of MCI in veterans with diabetes, high cholesterol, obesity, who are frequent alcohol consumers and physically inactive which could help clarify some of the findings in this study. The implications of these findings support efforts by UK government bodies to continually promote a ‘get active’ public health message for the UK population. Promoting a healthier lifestyle and engagement in activities to prevent declining CVH could have positive long-term benefits in prevention against MCI and dementia [35]. Continued efforts are still required to ensure veterans register with their local General Practitioner which could provide the opportunity to reiterate the importance of frequent health checks to detect early signs of declining CVH, provide advice on how to maintain a healthy lifestyle and how to access specialist care to support a healthy lifestyle.

In summary, further work is required to understand the differences in the risk of MCI in non-veterans and veterans with obesity, in those who frequently consume alcohol, or were physically inactive. Within veterans, monitoring those with diabetes or high cholesterol may play a key role in delaying the onset and progress of MCI.

## Supplementary Material

kqae027_suppl_Supplementary_Material

## References

[1] Petersen R. Mild cognitive impairment. Continuum (Minneap Minn). 2016;22:404–418. doi:10.1586/14737175.2013.856265 27042901 PMC5390929

[2] Livingston G , Sommerlad A, Orgeta V et al. Dementia prevention, intervention, and care. Lancet 2017;390:2673–2734. 28735855 10.1016/S0140-6736(17)31363-6

[3] Rosenberg A , Ngandu T, Rusanen M, et al. Multidomain lifestyle intervention benefits a large elderly population risk for cognitive decline and dementia regardless of baseline characteristics: the FINGER trial. Alzheimers Dementia 2018;14:263–270. 10.1016/j.jalz.2017.09.006 29055814

[4] Ciobica A , Padurariu M, Bild W, Stefanescu C. Cardiovascular risk factors as potential markers for mild cognitive impairment and Alzheimer’s disease. Psychiatr Danub 2011;23:340–346. 22075734

[5] Ngandu T , Lehtisalo J, Solomon A et al. A 2 year multidomain intervention of diet, exercise, cognitive training, and vascular risk monitoring versus control to prevent cognitive decline in at-risk elderly people (FINGER): a randomised controlled trial. Lancet 2015;385:2255–2263. 25771249 10.1016/S0140-6736(15)60461-5

[6] Samieri C , Perier M, Gaye B et al. Association of cardiovascular health level in older age with cognitive decline and incident dementia. JAMA 2018;320:657–664. 30140876 10.1001/jama.2018.11499 PMC6142948

[7] The British Army. *Army Medical Requirements*. https://jobs.army.mod.uk/how-to-join/can-i-apply/medical/?_gl=1*1cdqzu5*_ga*MTA5NjkwMjEyNi4xNjkyMzYyODE4*_ga_GBLXYTKLHV*MTY5MjM2MjgxOC4xLjAuMTY5MjM2MjgxOC4wLjAuMA..#which-medical-conditions-will-stop-me-joining? (18 August 2023, date last accessed).

[8] Veitch D , Friedl K, Weiner M. Military risk factors for cognitive decline, dementia and Alzheimer’s disease. Curr Alzheimer Res 2013;10:907–930. 23906002 10.2174/15672050113109990142

[9] Williamson V , Rossetto A, Murphy D. Relationship between obesity and health problems in help-seeking veterans. BMJ Mil Heal 2020;166:227–231. 10.1136/jramc-2019-001155 30709923

[10] Hinojosa R. Cardiovascular disease among United States military veterans: evidence of a waning healthy soldier effect using the National Health Interview Survey. Chronic Illn 2020;16:55–68. 29940779 10.1177/1742395318785237

[11] Ministry of Defence. *UK Armed Forces Veterans Residing in Great Britain, 2017: Annual Population Survey*. https://assets.publishing.service.gov.uk/government/uploads/system/uploads/attachment_data/file/774937/20190128_-_APS_2017_Statistical_Bulletin_-_OS.pdf (31 March 2022, date last accessed).

[12] Chen M , Hu C, Dong H, Yan H, Wu P; Alzheimer’s Disease Neuroimaging Initiative. A history of cigarette smoking is associated with faster functional decline and reduction of entorhinal cortex volume in mild cognitive impairment. Aging (Albany NY) 2021;13:6205–6213. 33578392 10.18632/aging.202646 PMC7950256

[13] Littman AJ , Forsberg CW, Koepsell TD. Physical activity in a national sample of veterans. Med Sci Sports Exerc 2009;41:1006–1013. 19346987 10.1249/MSS.0b013e3181943826

[14] Müller J , Chan K, Myers JN. Association between exercise capacity and late onset of dementia, Alzheimer disease, and cognitive impairment. Mayo Clin Proc 2017;92:211–217. 28082018 10.1016/j.mayocp.2016.10.020

[15] Huntley J , Corbett A, Wesnes K et al. Online assessment of risk factors for dementia and cognitive function in healthy adults. Int J Geriatr Psychiatry 2018;33:e286–e293. 28960500 10.1002/gps.4790

[16] Stevelink SAM , Jones M, Hull L et al. Mental health outcomes at the end of the British involvement in the Iraq and Afghanistan conflicts: a cohort study. Br J Psychiatry 2018;213:690–697. 30295216 10.1192/bjp.2018.175 PMC6429255

[17] Burdett H , Woodhead C, Iversen AC, et al. ‘Are you a veteran?’ understanding of the term ‘veteran’ among UK ex-service personnel: a research note. Armed Forces Soc 2012;39:751–759.

[18] National Health Service. *Obesity*. 2023. https://www.nhs.uk/conditions/obesity/#:~:text=18.5%20to%2024.9%20%E2%80%93%20you’re,in%20the%20severely%20obese%20range. (2 June 2023, date last accessed).

[19] Winblad B , Palmer K, Kivipelto M et al. Mild cognitive impairment—beyond controversies, towards a consensus: report of the International Working Group on Mild Cognitive Impairment. J Intern Med 2004;256:240–246. 15324367 10.1111/j.1365-2796.2004.01380.x

[20] Jorm AF. The Informant Questionnaire on Cognitive Decline in the Elderly (IQCODE): a review. Int Psychogeriatr 2004;16:275–293. 15559753 10.1017/s1041610204000390

[21] Desai R , Charlesworth G, Brooker H, et al. Temporal relationship between depressive symptoms and cognition in mid and late life: a longitudinal cohort study. J Am Med Dir Assoc 2020;21:1108–1113. 32151550 10.1016/j.jamda.2020.01.106

[22] Lang AJ , Stein MB. An abbreviated PTSD checklist for use as a screening instrument in primary care. Behav Res Ther 2005;43:585–594. 15865914 10.1016/j.brat.2004.04.005

[23] Spitzer RL , Kroenke K, Williams JB, Löwe B. A brief measure for assessing generalized anxiety disorder: the GAD-7. Arch Intern Med 2006;166:1092–1097. 16717171 10.1001/archinte.166.10.1092

[24] Kroenke K , Spitzer RL, Williams JB. The PHQ-9: validity of a brief depression severity measure. J Gen Intern Med 2001;16:606–613. 11556941 10.1046/j.1525-1497.2001.016009606.x PMC1495268

[25] Strand LA , Martinsen JI, Fadum EA, Borud EK. Temporal trends in the healthy soldier effect in a cohort of Royal Norwegian Navy servicemen followed for 67 years. Occup Environ Med 2020;77:775–781. 32611649 10.1136/oemed-2020-106475

[26] Åberg MAI , Pedersen NL, Torén K et al. Cardiovascular fitness is associated with cognition in young adulthood. Proc Natl Acad Sci USA 2009;106:20906–20911. 19948959 10.1073/pnas.0905307106 PMC2785721

[27] Andel R , Crowe M, Pedersen NL, Fratiglioni L, Johansson B, Gatz M. Physical exercise at midlife and risk of dementia three decades later: a population-based study of Swedish twins. J Gerontol A Biol Sci Med Sci 2008;63:62–66. 18245762 10.1093/gerona/63.1.62

[28] ForcesWatch. *ForcesWatch Briefing: The Recruitment of Under 18s into the UK Armed Forces*. 2011. https://www.parliament.uk/globalassets/documents/joint-committees/human-rights/Briefing_from_Forces_Watch_age_of_recruitment.pdf

[29] Centres for Disease Control and Prevention. *Body mass index: consideration for practitioners*. Published August 2011. https://stacks.cdc.gov/view/cdc/25368

[30] Valenza S , Paciaroni L, Paolini S et al. Mild cognitive impairment subtypes and type 2 diabetes in elderly subjects. J Clin Med 2020;9:2055. 32629878 10.3390/jcm9072055 PMC7408775

[31] Anstey KJ , Ashby-Mitchell K, Peters R. Updating the evidence on the association between serum cholesterol and risk of late-life dementia: review and meta-analysis. J Alzheimers Dis 2017;56:215–228. 27911314 10.3233/JAD-160826 PMC5240556

[32] Glovaci D , Fan W, Wong ND. Epidemiology of diabetes mellitus and cardiovascular disease. Curr Cardiol Rep 2019;21:21. 30828746 10.1007/s11886-019-1107-y

[33] Mortensen MB , Nordestgaard BG. Elevated LDL cholesterol and increased risk of myocardinal infarction and atherosclerotic cardiovascular disease in individuals aged 70-100 years: a contemporary primary prevention cohort. Lancet 2020;396:1644–1652. 33186534 10.1016/S0140-6736(20)32233-9

[34] Zhang Y , Vittinghoff E, Pletcher MJ et al. Associations of blood pressure and cholesterol levels during young adulthood with later cardiovascular events. J Am Coll Cardiol 2019;74:330–341. 31319915 10.1016/j.jacc.2019.03.529 PMC6764095

[35] Kivipelto M , Ngandu T, Laatikainen T, Winblad B, Soininen H, Tuomilehto J. Risk score for the prediction of dementia risk in 20 years among middle aged people: a longitudinal, population-based study. Lancet Neurol 2006;5:735–741. 16914401 10.1016/S1474-4422(06)70537-3

