# Supplementary material for: Cardiovascular and lifestyle risk factors of mild cognitive impairment in UK veterans and non-veterans
Source: Occup Med (Lond). 2024 Jun 11;74(4):274–82. doi: 10.1093/occmed/kqae027 (PMC11165370; doi:10.1093/occmed/kqae027)
Supplement: kqae027_suppl_Supplementary_Material [file kqae027_suppl_supplementary_material.docx]

**Supplementary A:** Military Service History Questionnaire

Military Service History Survey

Please circle/tick where appropriate, thank you.

1. Are you a carer or close relative of someone who has served in the UK Armed Forces (Royal Navy, Royal Marines, British Army or Royal Air Force)?
2. Yes
   1. If so, please could you describe what relationship you have with someone who has served in the UK Armed Forces. “I am their…

| Spouse/ Partner | □ | Parent | □ | | Child | □ |
| --- | --- | --- | --- | --- | --- | --- |
| Sibling | □ | Carer | | □ |  | □ |
| Other (please specify)………………………………………………………..... □ | | | | | |  |

1. No
2. Have you ever served in the UK Armed Forces (as a regular or reserve)?
3. Yes
4. No (do not answer any further questions; thank you for your participation)
5. Which branch of the UK Armed Forces do/ did you serve in? (please circle all that apply)
6. British Army
7. Royal Navy
8. Royal Marines
9. Royal Air Force
10. What year did you first serve in the UK Armed Forces? (Please enter the year as numbers e.g. 1990)

a…………………………

1. What year did you last serve in the UK Armed Forces? (Please enter the year as numbers e.g. 1990)

a………………………

b. I am still serving

1. Are you or were you a regular, reserve or have you served as both a regular and as a reserve?
2. Regular
3. Reserve
4. Both regular and reserve
5. Other, please specify

| Royal Navy | | Army and Royal Marines | | | RAF | |
| --- | --- | --- | --- | --- | --- | --- |
| AB | □ | Pte/Mne | □ | | AC/LAC/SAC/JT | □ |
| LH/LR | □ | LCpl to Cpl | | □ | Cpl | □ |
| PO to WO1 | □ | Sgt to WO1 | | □ | Sgt to WO | □ |
| Mid to Lt Cdr | □ | 2^nd^ Lt to Maj | | □ | Plt Off to Sqn Ldr | □ |
| Cdr & above | □ | Lt Col & above | | □ | Wg Cdr & above | □ |
| Other (please specify)………………….. | □ | Other (please specify)……… | | □ | Other (please specify)……… | □ |

1. What is/ was your rank when you left the UK Armed Forces? (please tick)
2. What is/was your primary role/trade within your parent unit? (please tick)

| Combat | □ | Medical | □ | | Engineering | □ |
| --- | --- | --- | --- | --- | --- | --- |
| Communications | □ | Warfare | | □ | Intelligence | □ |
| Policing | □ | Aircrew | | □ | Flight operations | □ |
| Training / education | □ | Logistics (including driver, catering, supply) | | | | □ |
| Other (please specify)………………………………………………………..... □ | | | | | |  |

1. Has a traumatic event or experience ever happened to you at any time in your life? (The term traumatic event or experience means something like a major natural disaster, a serious automobile accident, being raped, seeing someone killed or seriously injured, having a loved one die by murder or suicide, or any other experience that either put you or someone close to you at risk of serious harm or death.)
2. Yes
3. No (skip to Q10)
4. Don't understand/does not apply
5. Have the traumatic experiences you have been exposed to been...
6. Directly related to your military career
7. Not related to your military career
8. Or both?
9. During your service in the UK Armed Forces, have/ did you deploy on operational duties (rather than on exercise)?
10. Yes (please answer Q11)
11. No
12. If so, please specify where you were deployed and the number of deployments:

| **Deployment name (if known)** | **Country/Location** | **Years of deployment (provide multiple if appropriate)** |
| --- | --- | --- |
|  |  |  |
|  |  |  |
|  |  |  |
|  |  |  |
|  |  |  |

Thank you for taking the time to complete this survey.
